# Zebrafish-Based Discovery of Antiseizure Compounds from the North Sea: Isoquinoline Alkaloids TMC-120A and TMC-120B

**DOI:** 10.3390/md17110607

**Published:** 2019-10-25

**Authors:** Daniëlle Copmans, Sara Kildgaard, Silas A. Rasmussen, Monika Ślęzak, Nina Dirkx, Michèle Partoens, Camila V. Esguerra, Alexander D. Crawford, Thomas O. Larsen, Peter A. M. de Witte

**Affiliations:** 1 Laboratory for Molecular Biodiscovery, Department of Pharmaceutical and Pharmacological Sciences, KU Leuven, Herestraat 49 box 824, 3000 Leuven, Belgium; danielle.copmans@kuleuven.be (D.C.); monika.slezak@wp.pl (M.Ś.); Nina.Dirkx@uantwerpen.be (N.D.); michele.partoens@kuleuven.be (M.P.); c.v.esguerra@farmasi.uio.no (C.V.E.); alexander.dettmar.crawford@nmbu.no (A.D.C.); 2 Department of Biotechnology and Biomedicine, Technical University of Denmark, Søltofts Plads, Building 221, 2800 Kgs. Lyngby, Denmark; sarakildgaard@gmail.com (S.K.); silas.anselm@gmail.com (S.A.R.); 3 Current affiliation: Centre for Molecular Medicine Norway, Faculty of Medicine, University of Oslo, Gaustadalléen 21, 0349 Oslo, Norway; 4 Current affiliation: Faculty of Veterinary Medicine, Norwegian University of Life Sciences, Ullevålsveien 72, 0454 Oslo, Norway

**Keywords:** PharmaSea, epilepsy, zebrafish, marine drug discovery, isoquinoline alkaloids, TMC-120A, TMC-120B, TMC-120C, penicisochroman G, ustusorane B

## Abstract

There is a high need for the development of new and improved antiseizure drugs (ASDs) to treat epilepsy. Despite the potential of marine natural products (MNPs), the EU marine biodiscovery consortium PharmaSea has made the only effort to date to perform ASD discovery based on large-scale screening of MNPs. To this end, the embryonic zebrafish photomotor response assay and the larval zebrafish pentylenetetrazole (PTZ) model were used to screen MNP extracts for neuroactivity and antiseizure activity, respectively. Here we report the identification of the two known isoquinoline alkaloids TMC-120A and TMC-120B as novel antiseizure compounds, which were isolated by bioactivity-guided purification from the marine-derived fungus *Aspergillus insuetus*. TMC-120A and TMC-120B were observed to significantly lower PTZ-induced seizures and epileptiform brain activity in the larval zebrafish PTZ seizure model. In addition, their structural analogues TMC-120C, penicisochroman G, and ustusorane B were isolated and also significantly lowered PTZ-induced seizures. Finally, TMC-120A and TMC-120B were investigated in a mouse model of drug-resistant focal seizures. Compound treatment significantly shortened the seizure duration, thereby confirming their antiseizure activity. These data underscore the possibility to translate findings in zebrafish to mice in the field of epilepsy and the potential of the marine environment for ASD discovery.

## 1. Introduction

Marine drug discovery has only been systematically performed since the 1970s, once scuba diving could be used routinely [1]. The interest to develop drugs of marine origin comes from the fact that marine species could produce unique secondary metabolites in terms of chemistry and bioactivity, as these organisms have evolved to survive under extreme conditions, i.e., of darkness, salinity, pressure, and temperature [2,3,4]. Moreover, oceans cover more than 70% of the world surface and are largely unexplored due to the difficulty to access marine species in general and deep-sea life specifically [5,6]. 

PharmaSea was a European marine biodiscovery project that was funded by the Seventh Framework Programme (FP7). The project consisted of 24 partners from SME and academia from 13 different countries [3,7]. From 2012 to 2017, PharmaSea sought to systematically identify novel antibiotic, anti-inflammatory, and neuroactive compounds from marine microorganisms that were isolated from a variety of ocean environments, with a focus on deep and cold-sea biomes [3,7]. Neuroactive marine natural product (MNP) discovery within PharmaSea focused on the identification of compounds with the potential to treat epilepsy.

Epilepsy is among the most common severe neurological conditions, affecting more than 70 million people worldwide [8,9,10]. It is characterized by an enduring predisposition of the brain to generate epileptic seizures, with neurobiological, cognitive, psychological, and social consequences [11]. The treatment of epilepsy consists mostly of pharmacotherapy with antiseizure drugs (ASDs) to control seizures [12]. Despite the availability of more than 25 approved ASDs, these drugs fail to control seizures in approximately 30% of epilepsy patients due to drug-resistance [13]. Uncontrolled epilepsy can result in a poorer quality of life because of physical, psychological, cognitive, social, and occupational problems [12,14]. Moreover, first-line ASDs are associated with important adverse effects that can significantly impact daily life and are an important cause of treatment failure [13,15,16]. Hence, there is a significant need for the development of safer ASDs that are more effective against drug-resistant seizures [17,18]. 

ASD discovery efforts have been dominated to date by the use of rodent seizure and epilepsy models [19,20]. However, these models are not suitable for large-scale drug screening as they are too labor-intensive and time- and resource-consuming. Moreover, there are significant ethical concerns associated with rodents and therefore their use is subject to strict regulations [21,22,23]. Another disadvantage of rodents for marine drug discovery is that the quantity of MNPs for primary screening purposes is often too limited. Zebrafish represent an attractive alternative for high-throughput screening of MNPs as they require only sub-milligram quantities for hit selection and validation and are amenable to multi-well plates given the small size of their embryos and larvae [21,24,25]. Like rodents, zebrafish capture much of the complexity of vertebrate physiology including the central nervous system, and can be used for phenotype-based drug discovery which allows the identification of bioactive compounds independently of their mode of action [24,25]. 

For these reasons, we used embryonic and larval zebrafish bioassays as an initial screening platform to identify MNPs with antiseizure activity prior to further validation and characterization in rodent seizure models. Towards this end, the zebrafish photomotor response (PMR) assay and the zebrafish pentylenetetrazole (PTZ) seizure model were used to identify neuroactive and antiseizure hits, respectively. The PMR is a stereotypical behavior of 30–40 h post-fertilization (hpf) zebrafish embryos that is triggered by two subsequent high-intensity light pulses [26,27]. It was reported as a robust behavior that is useful for high-throughput neuroactive drug discovery [26], which was confirmed by our group in an independent study [28]. The larval zebrafish PTZ seizure model is a chemically-induced seizure model that relies on the GABA_A_-R antagonist PTZ that is well-known to induce generalized seizures in rodent seizure models [29,30]. The larval zebrafish PTZ seizure model was chosen as (1) it is validated for the presence of behavioral and non-behavioral seizure biomarkers, (2) has been pharmacologically characterized—showing not only a high level of translation to rodent models but also the capacity to identify a broad range of ASDs with diverse MOAs—and (3) is suitable for high-throughput screening [29,31,32]. In this study we report the successful use of our zebrafish-based screening approach to identify the two known isoquinoline alkaloids TMC-120A and TMC-120B as new antiseizure compounds. TMC-120A and TMC-120B were isolated from the marine-derived fungus *Aspergillus insuetus* IBT 28443, which was collected from a seawater trap set in the North Sea, in between Denmark and Norway. This also led to the isolation of the structural analogues TMC-120C, penicisochroman G, and ustusorane B. These compounds are proposed as potential ASD leads that are worth further investigation for the treatment of epileptic seizures.

## 2. Results and Discussion

### 2.1. Zebrafish-Based Antiseizure Drug Discovery

**Figure 1:**
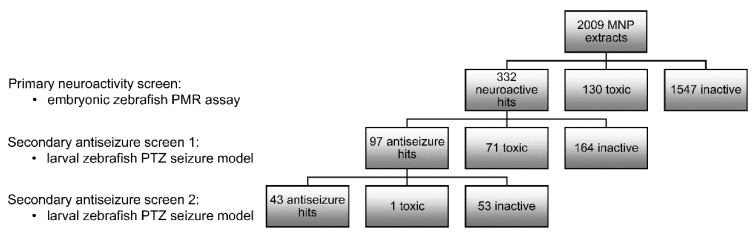
Zebrafish-based identification of neuroactive and antiseizure marine natural product (MNP) extracts. MNP extracts (crude extracts and fractions of pre-fractionated extracts) underwent a primary screen for neuroactivity at 100 µg/mL (2-h incubation time, *n* = 1), using the embryonic zebrafish photomotor response (PMR) assay, which was followed immediately after by a toxicity evaluation. Neuroactive hits (non-toxic) underwent a secondary screen for antiseizure activity at 100 µg/mL (2-h incubation time, *n* = 3), using the larval zebrafish pentylenetetrazole (PTZ) seizure model, which was followed immediately after by a toxicity evaluation. Antiseizure hits (non-toxic) underwent a second round of antiseizure analysis at 100, 33, and 11 µg/mL (2-h incubation time, *n* = 6) using the larval zebrafish PTZ seizure model, which was followed immediately after by a toxicity evaluation.

Over 2000 MNP extracts, including both crude extracts and fractions of pre-fractionated extracts, provided by the different PharmaSea partners, were screened for neuroactivity at a concentration of 100 µg/mL (2-h incubation time) using the zebrafish PMR assay (Figure 1). The PMR was described by a behavioral fingerprint of 16 pseudo Z-scores that represent the embryonic motion over a 30 s recording period using the first and third quantile (Q1 and Q3) for each of the eight time periods, as previously reported [28]. A neuroactive hit was defined as an MNP that modified the PMR such that its behavioral fingerprint contained at least one pseudo Z-score with an absolute value equal to or exceeding 5. Each PMR-assay was followed by visual evaluation of the embryos under a light microscope to assess toxicity of treatment. Only 130 MNPs were observed to cause toxicity at this concentration. All other treatments did not induce toxicity under the test conditions, whereof 332 were neuroactive and 1547 samples were inactive. The 332 neuroactive hits underwent antiseizure analysis at a concentration of 100 µg/mL (2-h incubation time) using the zebrafish PTZ seizure model (Figure 1). In this model the convulsant PTZ (20 mM) is administered to the swimming water of 7-days post-fertilization (dpf) larvae and induces typical seizure-like behavior that is characterized by high-speed swimming, whirlpool-like circling, clonus-like seizures, and loss of posture, as previously described [29]. An antiseizure hit was defined as an MNP that significantly lowered the strongly elevated larval locomotion as a result of PTZ-induced seizures. Initially, 97 antiseizure hits were identified that did not result in toxicity, whereof 43 were confirmed in a second screen using twice the number of larvae per sample. Moreover, the latter screen investigated concentration-dependent effects by analyzing a three-fold serial dilution from 100 µg/mL onwards (Figure 1). Hit prioritization was based on efficacy, concentration-dependency, and sample availability. 

**Figure 2:**
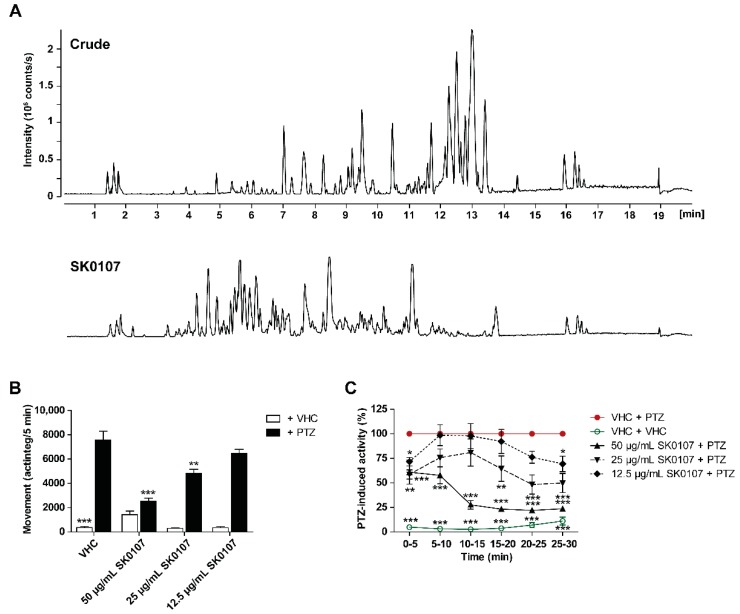
Antiseizure hit SK0107. (**A**) *Aspergillus insuetus* IBT 28443 cultivated on czapek yeast extract agar (CYA) and yeast extract sucrose agar (YES) media for 9 days at 25 °C in the dark. Base peak chromatograms of the crude extract and bioactive fraction SK0107 in positive electrospray ionization mode. (**B**,**C**) Antiseizure activity of SK0107 in the zebrafish pentylenetetrazole (PTZ) seizure model after 2 h of incubation. PTZ-induced seizure-like behavior is expressed as mean actinteg units per 5 min (±SEM) during the 30-min recording period (**B**) and over consecutive time intervals (**C**). Means are pooled from three independent experiments with each 12 replicate wells per condition. Statistical analysis: (**B**) one-way ANOVA with Dunnett’s multiple comparison test, (**C**) two-way ANOVA with Bonferroni posttests (GraphPad Prism 5, San Diego, CA, USA). Significance levels: * *p* ≤ 0.05; ** *p* ≤ 0.01; *** *p* ≤ 0.001. Abbreviation: vehicle, VHC.

Among prioritized hits was MNP SK0107, one of the more polar fractions from initial reversed phase chromatographic separation of the crude extract of *Aspergillus insuetus* IBT 28443 (Figure 2A), which was isolated from a seawater trap set in the North Sea, in between Norway and Denmark. *Aspergillus insuetus* is a filamentous fungus belonging to the *Aspergillus* section *Usti* that includes species from soil, foods, and indoor air environments [33] but also from marine isolates [34]. Marine-derived fungal isolates, with *Aspergillus* species as a common source, have been seen to yield a plethora of biologically active compounds [34]. Prior to further experiments the maximum tolerated concentration (MTC) of SK0107 was determined, which was defined as the highest concentration at which no larvae died nor showed signs of toxicity or locomotor impairment in comparison to vehicle (VHC)-treated control larvae. The MTC was observed to be 50 µg/mL and used as the highest test concentration in all subsequent tests. To validate the results obtained during the course of screening, the antiseizure activity of SK0107 was investigated in the larval zebrafish PTZ seizure model at the MTC, MTC/2, and MTC/4 (two-fold serial dilution, 2-h incubation time) in three independent experiments (Figure 2B,C). In line with former results, the antiseizure hit SK0107 showed significant concentration-dependent activity against PTZ-induced seizure behavior, both during the 30-min recording period (*p* ≤ 0.001 and *p* ≤ 0.01) (Figure 2B) as over consecutive 5-min time intervals (*p* ≤ 0.001, *p* ≤ 0.01, and *p* ≤ 0.05) (Figure 2C). 

### 2.2. Bioactivity-Guided Purification of Active Compounds

To identify the active constituents of SK0107 that were responsible for its antiseizure activity, bioactivity-guided purification was performed on the marine-derived fungus *Aspergillus insuetus* IBT 28443. In the crude extract of *Aspergillus insuetus* dereplication using ultra-high performance liquid chromatography–diode array detection–high resolution mass spectrometry (UHPLC–DAD–HRMS) tentatively identified an abundant presence of the sesterterpenoids, ophiobolins [35] (inactive, data not shown). Before any large-scale cultivation, small-scale extracts were prepared of the fungus cultivated individually on czapek yeast extract agar (CYA), yeast extract sucrose agar (YES), and oatmeal agar (OAT) media, as the tested bioactive extract was of the combined cultivation on both CYA and YES media. This was done in the hope of finding a medium where the production of ophiobolins was reduced and other compounds presented in a higher concentration than the original crude extract. CYA medium was chosen based on the bioactivity of fractions from the crude extract and based on the reduced concentration of ophiobolins (data not shown). 

**Figure 3:**
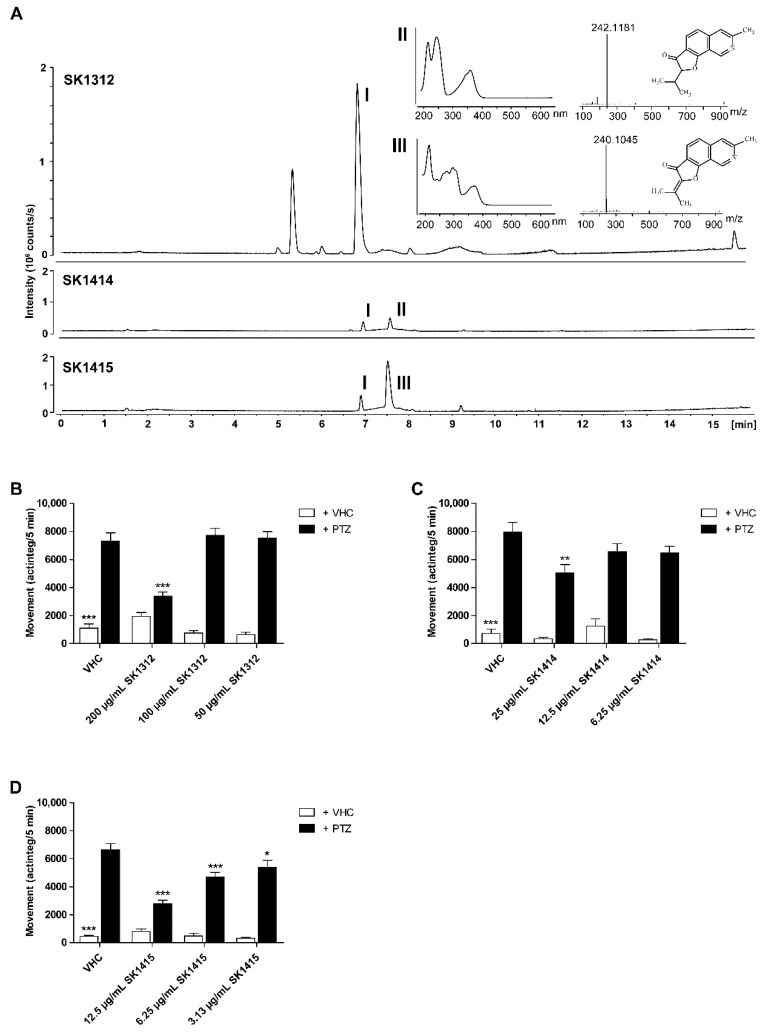
Bioactivity-guided identification of the active compounds of antiseizure hit SK0107. (**A**) *Aspergillus insuetus* IBT 28443 cultivated on czapek yeast extract agar (CYA) media for 9 days in the dark at 25 °C. Base peak chromatogram (BPC) of the most bioactive fraction (SK1312) from first reversed-phase fractionation in positive electrospray ionization mode (ESI^+^). ESI^+^ BPC chromatograms of the two most bioactive fractions (SK1414 and SK1415) from the second reversed-phase fractionation. UV/Vis and HRMS spectra for TMC-120A (II) and TMC-120B (III). (**B**–**D**) Antiseizure activity of SK1312 (*n* = 23–24 replicate wells per condition) (**B**), SK1414 (*n* = 10–11 replicate wells per condition) (**C**), and SK1415 (*n* = 22 replicate wells per condition) (**D**) in the zebrafish pentylenetetrazole (PTZ) seizure model after 2 h of incubation at their maximum tolerated concentration (MTC), MTC/2, and MTC/4. PTZ- induced seizure-like behavior is expressed as mean actinteg units per 5 min (±SEM) during the 30-min recording period. (**B**,**D**) Data are pooled from two independent experiments with each 11–12 replicate wells per condition. (**C**) Data are from a single experiment with 10–11 replicate wells per condition. (**B**–**D**) Statistical analysis: one-way ANOVA with Dunnett’s multiple comparison test for comparison of sample + PTZ groups with vehicle (VHC) + PTZ control group, Kruskal–Wallis test with Dunn’s multiple comparison test (data did not pass the Shapiro–Wilk normality test) for comparison of sample + VHC groups with VHC + VHC control group (GraphPad Prism 5, San Diego, CA, USA). Significance levels: * *p* ≤ 0.05; ** *p* ≤ 0.01; *** *p* ≤ 0.001.

**Figure 4:**
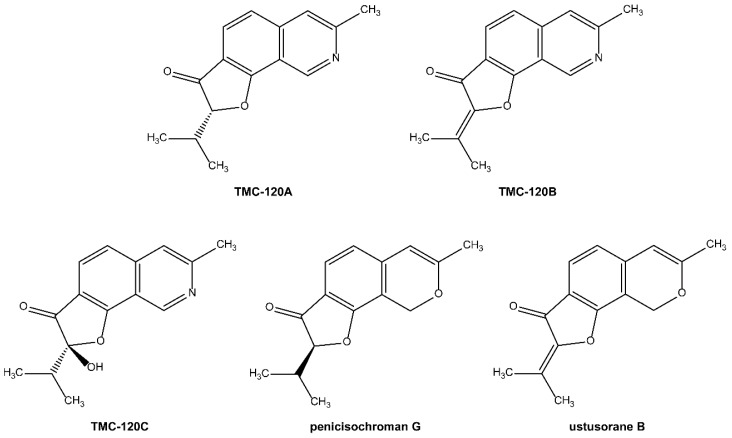
Chemical structures of the isoquinoline alkaloids TMC-120A and TMC-120B and structural analogues TMC-120C, penicisochroman G, and ustusorane B.

A large-scale extract was prepared from the cultivation of *Aspergillus insuetus* IBT 28443 on CYA media for 9 days in the dark at 25 °C and bioactivity-guided purification was performed through several reversed-phase purification steps until single compound isolation. In the two most bioactive fractions from the second reversed-phase fractionation of the crude extract, i.e., SK1414 and SK1415, two related compounds were tentatively identified by UHPLC–DAD–HRMS dereplication (Figure 3). The compounds were observed to co-elute by first fractionation with the pseudomolecular ions, [M + H]^+^ *m/z* 242.1177 (mass accuracy −0.32 ppm) and *m/z* 240.1019 (accuracy 0.14 ppm). The molecular formulas for the two compounds were based on the pseudomolecular ions established to be C_15_H_15_NO_2_ and C_15_H_13_NO_2_ respectively. A search in Antibase 2012 [36] for the formulas revealed the possible candidates to be the isoquinoline alkaloids TMC-120A and TMC-120B (Figure 3 and Figure 4). This was supported by UV/Vis data consistent with literature, with both compounds displaying characteristic UV/Vis spectra, and production by related fungal species (*Aspergillus ustus*) [37]. The structure of TMC-120B was confirmed by 1D and 2D NMR structural elucidation and comparison of ^1^H and ^13^C chemical shifts to literature data [37]. A third active compound was identified as well in the bioactive fractions (I, Figure 3), but as it belongs to a different compound class and was also studied in depth, this work will be published separately. 

**Figure 5:**
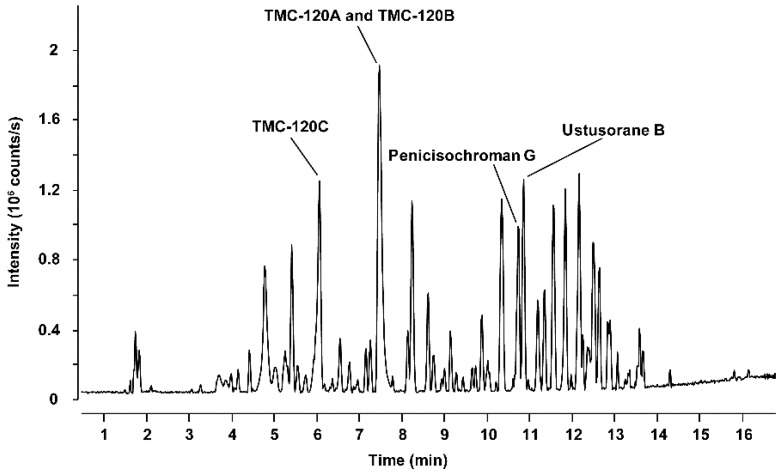
*Aspergillus insuetus* IBT 28485 cultivated on czapek yeast extract agar (CYA) media for 9 days in the dark at 25 °C. Base peak chromatogram of the crude extract with marked peaks for TMC-120A and TMC-120B, TMC-120C, penicisochroman G, and ustusorane B in positive electrospray ionization mode.

Only trace amounts of TMC-120A and TMC-120B (≤0.5 mg) could be purified from the crude extract of *Aspergillus insuetus* IBT 28443. Therefore, various closely related species belonging to *Aspergillus* section *Usti* (Table 1) were investigated by HRMS, MS/HRMS and UV/Vis data analysis to find a better fungal producer. *Aspergillus insuetus* IBT 28485 was chosen based on its production of TMC-120A and TMC-120B as some of the main compounds (Figure 5) and these were purified in higher amounts (≥10 mg). The structures of TMC-120A and TMC-120B were confirmed by 1D and 2D NMR structural elucidation and comparison of ^1^H and ^13^C chemical shifts and optical rotation to literature data [38]. Some of the other major metabolites of the fungus were identified to be structural analogues and were isolated, i.e., TMC-120C, penicisochroman G, and ustusorane B (Figure 4 and Figure 5), with HRMS, UV/Vis and ^1^H- and ^13^C-NMR data consistent with literature and in agreement with reported optical rotations (see data in Supplementary Materials) [37,38,39,40]. 

### 2.3. TMC-120A, TMC-120B, and Structural Analogues Ameliorate Seizures in the Zebrafish PTZ Seizure Model

**Figure 6:**
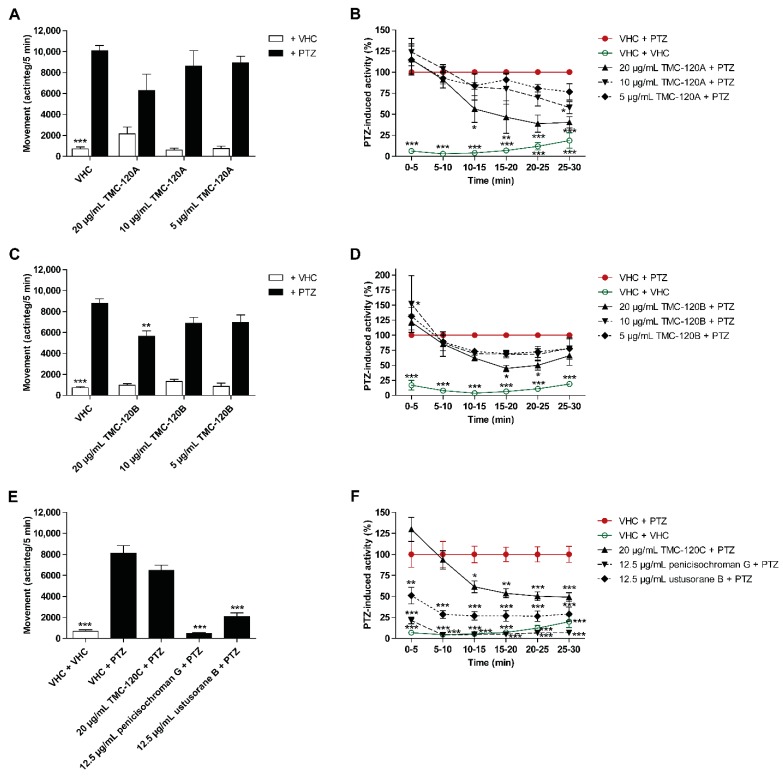
Behavioral antiseizure analysis of TMC-120A, TMC-120B, and structural analogues in the zebrafish PTZ seizure model. Antiseizure activity of TMC-120A (**A**,**B**), TMC-120B (**C**,**D**), and their structural analogues (TMC-120C, penicisochroman G, and ustusorane B) (**E**,**F**) in the zebrafish pentylenetetrazole (PTZ) seizure model after 2 h of incubation, respectively. PTZ-induced seizure-like behavior is expressed as mean actinteg units per 5 min (±SEM) during the 30-min recording period (**A**,**C**,**E**) and over consecutive time intervals (**B**,**D**,**F**). (**A**–**D**) Means are pooled from three independent experiments with each 10–12 replicate wells per vehicle (VHC) + PTZ and compound + PTZ condition, and 6–12 replicate wells per VHC + VHC and compound + VHC condition. (**E**–**F**) Data are pooled from two single experiments. Number of replicate wells per condition: 21–22 replicate wells for VHC + PTZ and VHC + VHC conditions, and 8–11 replicate wells for compound + PTZ conditions. Statistical analysis: (**A**,**C**,**E**) one-way ANOVA with Dunnett’s multiple comparison test, (**B**,**D**,**F**) two-way ANOVA with Bonferroni posttests (GraphPad Prism 5, San Diego, CA, USA). Significance levels: * *p* ≤ 0.05; ** *p* ≤ 0.01; *** *p* ≤ 0.001.

To confirm that TMC-120A and TMC-120B, isolated from the most bioactive fractions, are indeed the active constituents, their antiseizure activity was investigated in the zebrafish PTZ seizure model (Figure 6A–D). Larvae were treated with the compounds for 2 h, using their MTC, MTC/2, and MTC/4, conform with the conditions used for the crude extract and fractions. TMC- 120B, but not TMC-120A, significantly lowered PTZ-induced seizure behavior at its MTC during the 30-min recording period (*p* ≤ 0.01) (Figure 6A,C). A more detailed analysis of the behavior over consecutive 5-min time intervals revealed a significant reduction of PTZ-induced seizure behavior for both compounds at their MTCs: within the 10–30 min time window with *p* ≤ 0.05, *p* ≤ 0.01, and *p* ≤ 0.001 at different time intervals in case of TMC-120A, and within the 15–25 min time window with *p* ≤ 0.05 in case of TMC-120B (Figure 6B,D). No significant antiseizure activity was seen at lower concentrations, except for TMC-120A at the MTC/2 (*p* ≤ 0.05) in the last 5-min time interval. These data demonstrate the antiseizure activity of TMC-120A and TMC-120B and confirm that the isolated compounds are indeed active constituents of the antiseizure hit SK0107, and of the bioactive fractions SK1312, SK1414, and SK1415. The higher antiseizure efficacy of the bioactive extract and fractions in comparison to these observed for the individual compounds is possibly due to a synergistic or additive action, and/or to the presence of additional bioactive compounds. Synergism is commonly observed in natural product research and is proposed to be exploited in innovative combination drugs [41]. Nevertheless, the antiseizure efficacy of TMC-120A and TMC-120B is comparable to that of the positive control valproate (Figure S8 in Supplementary Materials), without the adverse observation of pronounced induced hyperactivity.

Interestingly, no antiseizure activity has yet been reported for TMC-120A and TMC-120B. Both compounds were first reported in 1999 by Kohno and colleagues, from an *Aspergillus ustus* TC 118 fermentation broth isolated from rhizosphere of grass [37,42], and were observed in vitro to have moderate anti-inflammatory activity against IL-5-mediated prolongation of eosinophil survival [42]. Although contradictory, TMC-120A was also shown to induce mild pro-inflammatory effects in the lungs of mice that underwent intratracheal instillation and to induce inflammation-associated gene modulation [43]. A follow-up in vitro study, using mouse alveolar macrophages, provided further support for these findings and suggested that oxidative stress can also be an important response to compound exposure [44]. In this study, TMC-120A and TMC-120B were identified as novel antiseizure compounds thereby broadening their potential therapeutic use to the field of neuroscience. Of note, despite the possibility to find novel metabolites when investigating marine-derived microorganisms, the identified active compounds are known and have been previously reported to be isolated from a terrestrial producing strain. This is in line with the fact that the selection of the active MNP extract, fractions, and compounds was purely based on their antiseizure activity and not on structural novelty.

The antiseizure activity of the structural analogues, TMC-120C, penicisochroman G, and ustusorane B, was investigated as well in the zebrafish PTZ seizure model after 2 h of incubation (Figure 6E,F). All three compounds lowered PTZ-induced seizure behavior at the tested concentration, which was significant for penicisochroman G (*p* ≤ 0.001) and ustusorane B (*p* ≤ 0.001) during the 30-min recording period (Figure 6E) and for all three compounds over consecutive 5-min time intervals (Figure 6F): within the 10–30 min time window for TMC-120C (*p* ≤ 0.05, *p* ≤ 0.01, and *p* ≤ 0.001) and over the entire recording period for penicisochroman G (*p* ≤ 0.001) and ustusorane B (*p* ≤ 0.01 and *p* ≤ 0.001) (Figure 6F). Hence, like TMC-120A and TMC120B, these analogues exhibit antiseizure activity as well and are worth further investigation for the treatment of epileptic seizures. Of note, these compounds were not further investigated in this study because of time constraints. 

TMC-120C was first discovered in 1999 by Kohno and colleagues, along with TMC-120A and TMC-120B, from an *Aspergillus ustus* TC 118 fermentation broth isolated from rhizosphere of grass [37,42]. In contrast to its analogues, TMC-120C did not show anti-inflammatory activity against IL-5-mediated prolongation of eosinophil survival [42]. Ustusorane B was first described in 2009 by Lu and colleagues from the EtOAc extract of the marine-derived fungus *Aspergillus ustus* 094102 isolated from the rhizosphere soil of a mangrove plant (*Bruguiera gymnorrhiza*) [38]. It was evaluated for cytotoxicity against cancer cell lines A549 and HL-60, but was found to be inactive [38]. Finally, the structure of penicisochroman G, along with ustusorane B, was published in 2014 by Bunbamrung and colleagues as a novel compound, obtained from the endophytic fungus *Penicillium* sp. BCC18034 [39]. Antimalarial activity against *P. falciparum* and cytotoxicity against KB, MCF-7, NCI-H187, and Vero cells were investigated. Penicisochroman G was inactive at the tested concentrations, while ustusorane B did show cytotoxicity against cancerous cells KB, MCF-7, and NCI-H187 at concentrations >10 µM [39].

### 2.4. TMC-120A and TMC-120B Ameliorate Epileptiform Brain Activity in the Zebrafish PTZ Seizure Model

**Figure 7:**
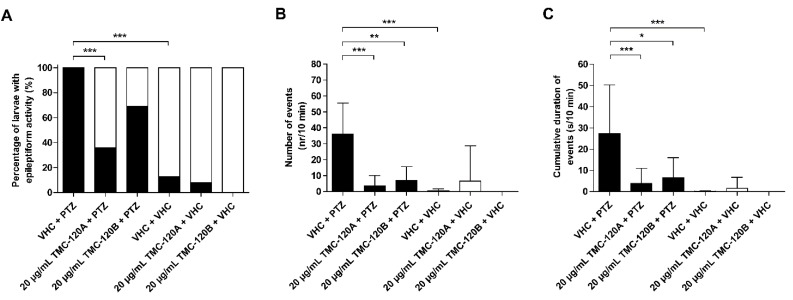
Electrophysiological antiseizure analysis of TMC-120A and TMC-120B in the zebrafish PTZ seizure model. Noninvasive local field potential recordings from the optic tectum of larvae pre-exposed to vehicle (VHC) and pentylenetetrazole (PTZ), VHC only, compound and PTZ, or compound and VHC. Larvae were incubated with 20 µg/mL TMC-120A or TMC-120B for 2 h, conform with the maximum tolerated concentrations and incubation time used in the behavioral assay. Larvae are considered to possess epileptiform brain activity when three or more epileptiform events occurred during a 10-min recording (**A**). Epileptiform discharges are quantified by the number (mean ±SD) (**B**) and cumulative duration (mean ± SD) (**C**) of events per 10-min recording. Number of replicate wells per condition: 19 larvae were used for VHC + PTZ controls, 16 larvae were used for VHC + VHC controls, 13–14 larvae were used for compound + PTZ conditions, and 12 larvae were used for compound + VHC conditions. Statistical analysis: (**A**) Fisher’s exact test with Bonferroni posttest, (**B**,**C**) Kruskal–Wallis test with Dunn’s multiple comparison test (data did not pass the Shapiro–Wilk normality test) (GraphPad Prism 5, San Diego, CA, USA). Significance levels: * *p* ≤ 0.05; ** *p* ≤ 0.01; *** *p* ≤ 0.001.

**Figure 8:**
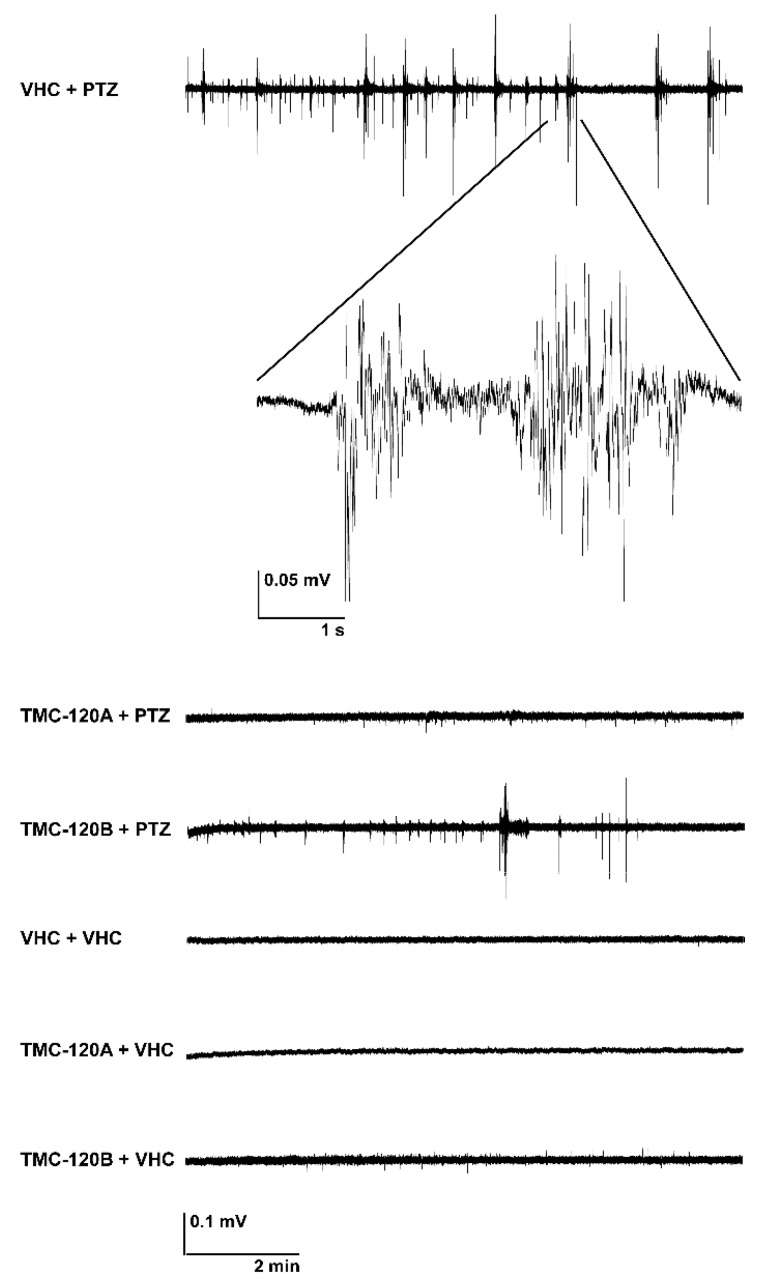
Representative local field potential recordings. 10-min noninvasive local field potential recordings from the optic tectum of larvae pre-exposed to vehicle (VHC) and pentylenetetrazole (PTZ), VHC only, compound and PTZ, or compound and VHC. Larvae were incubated with 20 µg/mL TMC-120A or TMC-120B for 2 h, conform with the maximum tolerated concentrations and incubation time used in the behavioral assay.

To determine whether TMC-120A and TMC-120B, in addition to their antiseizure activity, also ameliorate the PTZ-induced hyperexcitable state of the brain that is characterized by epileptiform discharges [45], local field potential (LFP) recordings [46] were non-invasively measured from the midbrain (optic tectum) of zebrafish larvae (Figure 7 and Figure 8). Larvae were treated with either VHC or compound (the MTC and a 2-h incubation time were used in line with previous experiments) followed by 15 min during exposure to PTZ or VHC prior to LFP measurements. In line with previous studies [7,29,32,46,47], pre-exposure to PTZ but not to VHC resulted in a significant increase of epileptiform electrical discharges. Pre-incubation with TMC-120A significantly lowered the percentage of larvae with PTZ-induced epileptiform activity by 64% (*p* ≤ 0.001) (Figure 7A). A larva was considered to have epileptiform brain activity when at least three electrical discharges were seen during the 10-min recording that fulfilled the pre-defined requirements of an epileptiform event (see Materials and Methods). In addition, pre-incubation with TMC-120A or TMC-120B significantly lowered the number (*p* ≤ 0.001 and *p* ≤ 0.01, respectively) and the cumulative duration (*p* ≤ 0.001 and *p* ≤ 0.05, respectively) of PTZ-induced epileptiform events over the 10-min recording period (Figure 7B,C). Thus, TMC-120A and TMC-120B show anti-epileptiform activity, which suggests that they display their antiseizure properties by counteracting the hyperexcitable state of the brain. The anti-epileptiform efficacy of TMC-120B is comparable to that of the positive control valproate and TMC-120A is even more effective than valproate (Figure S9 in Supplementary Materials).

### 2.5. TMC-120A and TMC-120B Ameliorate Focal Seizures in the Mouse 6-Hz (44 mA) Psychomotor Seizure Model

**Figure 9:**
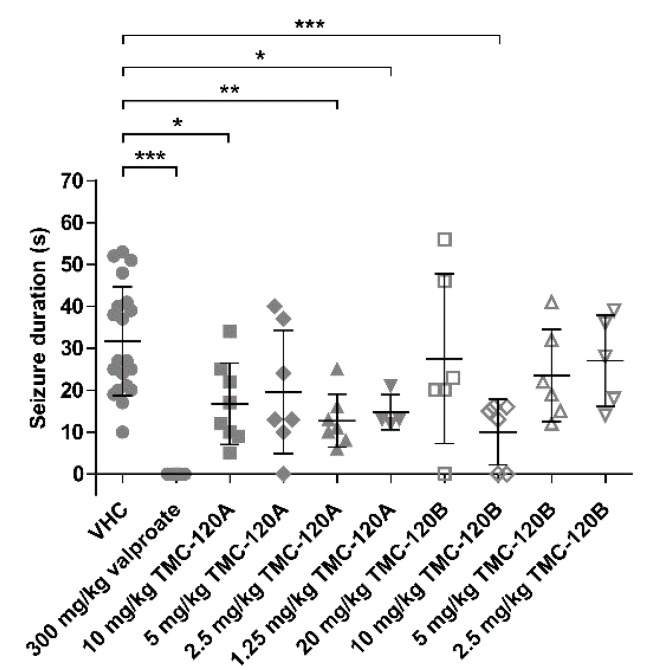
Antiseizure activity analysis of TMC-120A and TMC-120B in the mouse 6-Hz (44 mA) psychomotor seizure model. Drug-resistant psychomotor seizures were induced by electrical stimulation through the cornea, 30 min after i.p. injection of vehicle (VHC, *n* = 20), positive control valproate (*n* = 12), TMC-120A (*n* = 4–8) or TMC-120B (*n* = 5–6). Mean seizure durations (±SD) are depicted. Statistical analysis: one-way ANOVA with Dunnett’s multiple comparison test (GraphPad Prism 5, San Diego, CA, USA). Significance levels: * *p* ≤ 0.05; ** *p* ≤ 0.01; *** *p* ≤ 0.001.

Despite their high genetic, physiological, and pharmacological conservation, zebrafish are more distinct from humans than are mammals [24,48]. Therefore, we sought to investigate whether the antiseizure action of TMC-120A and TMC-120B observed in the larval zebrafish model translates to a standard rodent seizure model. From the available rodent seizure models, the mouse 6-Hz (44 mA) psychomotor seizure model was chosen. This is a widely accepted standard in current ASD discovery efforts that can detect compounds with novel antiseizure mechanisms and with potential activity against drug-resistant seizures [48,49,50]. It is an acute model of drug-resistant focal impaired awareness seizures [51], previously referred to as complex partial or psychomotor seizures [52], that are induced by a low-frequency, long-duration corneal electrical stimulation (6 Hz, 0.2 ms rectangular pulse width, 3 s duration, 44 mA) [50]. Seizures are characterized by a minimal clonic phase and stereotypical automatistic behaviors, typically seen as stun, forelimb clonus, Straub tail, and twitching of the vibrissae [49,53]. Male NMRI mice were intraperitoneally (i.p.) injected with VHC (DMSO:PEG200 1:1), positive control valproate (300 mg/kg), TMC-120A (10, 5, 2.5, and 1.25 mg/kg) or TMC-120B (20, 10, 5, and 2.5 mg/kg) 30 min before electrical stimulation (Figure 9). VHC injected mice showed characteristic seizure behavior with a mean (±SD) duration of 32 s (±13 s). In line with previous studies, valproate-treated mice were fully protected against the electrically-induced focal seizures [49,54] as none of the mice showed any seizure after electrical stimulation (*p* ≤ 0.001). Mice i.p. injected with TMC-120A had a shorter seizure duration than the VHC control group, which was significant at 10 mg/kg (*p* ≤ 0.05, mean duration of 17 s (±10 s)), 2.5 mg/kg (*p* ≤ 0.01, mean duration of 13 s (±6 s)), and 1.25 mg/kg (*p* ≤ 0.05, mean duration of 15 s (±4 s)), but not at 5 mg/kg (mean duration of 20 s (±15 s)). Mice i.p. injected with TMC-120B also had a shorter seizure duration than the VHC control group, which was significant at 10 mg/kg (*p* ≤ 0.001, mean duration of 10 s (±8 s)). A non-significant dose-dependent reduction in seizure duration was seen for mice injected with 5 and 2.5 mg/kg TMC-120B (mean duration of 23.5 s (±11 s) and 27 s (±11 s), respectively). Finally, at the higher dose of 20 mg/kg also a non-significant reduction in seizure duration was seen with a mean seizure duration of 27.5 s (±20 s). The latter effect can be influenced by the poor solubility of 20 mg/kg TMC-120B in the solvent in contrast to the lower doses. Thus, the antiseizure activity of TMC-120A and TMC-120B that was observed in the larval zebrafish PTZ seizure model translates to a standard mouse model of drug-resistant focal seizures. This demonstrates the effectiveness of the zebrafish-based ASD discovery approach and the potential of MNPs. Moreover, these observations also confirm the translation of findings from zebrafish larvae to mice in the field of epilepsy, as previously reported [7,53,55]. 

## 3. Materials and Methods 

### 3.1. General Chemical Experimental Procedures

UHPLC–DAD–HRMS was performed on an Agilent Infinity 1290 UHPLC system (Agilent Technologies, Santa Clara, CA, USA) which was equipped with a diode array detector. Separation was performed on an Agilent Poroshell 120 phenyl-hexyl column (2.1 × 150 mm, 2.7 µm). A linear gradient (0.35 mL/min, at 60 °C) was used of 10% acetonitrile (MeCN) in Milli-Q water buffered with 20 mM formic acid that was increased to 100% over 15 min, held for 2 min, returned to 10% in 0.1 min, and held for 3 min before the proceeding run. MeCN was LC-MS grade. MS detection was achieved on an Agilent 6550 iFunnel quadrupole time of flight MS equipped with an Agilent Dual Jet Stream electrospray ion source, with the drying gas temperature of 160 °C, gas flow of 13 L/min, sheath gas temperature of 300 °C and flow of 16 L/min. Capillary voltage was set to 4000 V and nozzle voltage to 500 V. Other MS parameters can be found described in Kildgaard et al. (2014) [35]. Data processing was performed using Agilent MassHunter Qualitative Analysis for quadrupole time of flight (version B.07.00). Elemental compositions of peaks corresponding to TMC-120A and TMC-120B and the structural analogues TMC-120C, penicisochroman G, and ustusorane B were identified based on mass accuracy, isotopic ratios, and isotopic pattern. Pre-fractionation was performed of the crude extracts by flash chromatography using an Isolera One automated flash system (Biotage, Uppsala, Sweden). Purification of active compounds was achieved using a Waters 600 Controller (Milford, MA, USA) that was coupled to a Waters 996 Photodiode Array Detector. 1D and 2D NMR experiments were acquired using standard pulse sequences on either a 400 MHz Bruker Ascend spectrometer with a Prodigy cryoprobe, a 600 MHz Bruker Ascend spectrometer with a SmartProbe (BBO) or a 800 MHz Bruker Avance spectrometer with a 5 mm TCI cryoprobe. Optical rotations were measured on a Perkin Elmer 341 polarimeter (Perkin Elmer, Waltham, MA, USA).

### 3.2. Fungal Strains

*Aspergillus insuetus* IBT 28443 and IBT 28485 were from the IBT culture collection at the Department of Biotechnology and Biomedicine, Technical University of Denmark. The marine-derived filamentous fungus *Aspergillus insuetus* IBT 28443 was collected at the Galathea 3 expedition [56] and isolated from a seawater trap set in the North Sea, in between Denmark and Norway (NMEA Latitude = 57 51.39 N, NMEA Longitude = 005 11.93 E, NMEA UCT (time) = Aug 13 2006 07:23:34).

### 3.3. Cultivation

*Aspergillus insuetus* IBT 28443 was cultivated on one CYA and one YES media plate for 9 days in the dark at 25 °C for the original combined small-scale cultivation. The CYA and YES plates were prepared as previously described [57]. For the individual small-scale cultivations the fungus was cultivated on eight plates of CYA, eight plates of YES and eight plates of OAT for 9 days in the dark at 25 °C. For the large-scale cultivation the fungus was cultivated on 250 plates of CYA for 9 days in the dark at 25 °C.

*Aspergillus insuetus* IBT 28485 was cultivated on 220 CYA plates for 7 days in the dark at 25 °C. 

### 3.4. Extraction and Isolation

For the original combined small-scale cultivation of *Aspergillus insuetus* IBT 28443, the two plates in total (one CYA and one YES) were extracted with 40 mL ethyl acetate (EtOAc) containing 1% formic acid. The EtOAc crude extract was fractionated on a reversed-phase C_18_ flash column (Sepra ZT, Isolute, 10 g) using the Isolera One automated flash system. The gradient used was 15% to 100% MeCN with a flow of 12 mL/min over 28 min and resulted in the automatic collection of 6 fractions based on the UV signal (210 nm and 254 nm). MeCN was of HPLC grade and the water was purified and deionized by a Millipore system (0.22 µm membrane filter), both MeCN and Milli-Q water were buffered with 20 mM formic acid. For the individual small-scale cultivations on CYA, YES, and OAT each of the separate set of eight plates was extracted with 150 mL EtOAc with 1% formic acid. For the large-scale cultivation on CYA, extraction was achieved with 150 mL EtOAc and 1% formic acid for every 10 plates. All the crude extracts were fractionated on a reversed-phase C_18_ flash column (Sepra ZT, Isolute, 25 g/33 mL) using the Isolera One automated flash system. The gradient used was 10% stepwise (12 column volumes) from 15% to 100% MeCN buffered with 20 mM formic acid and with a flow of 25 mL/min. Fractions were collected manually for every 10%. The most bioactive fraction (25% MeCN) of the large-scale cultivation was fractionated on a reversed phase Isolute SPE column (500 mg/3 mL) using methanol (MeOH) buffered with 20 mM formic acid. The metabolites were eluted with 2 column volumes per fraction: 15% MeOH, 20% MeOH, 30% MeOH, 40% MeOH, 50% MeOH, 60% MeOH, 80% MeOH, and 100% MeOH. TMC-120A and TMC-120B (60% MeOH and 80% MeOH) purification was achieved on a Gemini C_6_-Phenyl, 5 μm, 250 × 10 mm column (Phenomenex, Torrance, CA, USA) with a flow of 4 mL/min. A linear gradient was used of 40% MeCN in Milli-Q water with 20 mM formic acid going to 70% MeCN in 30 min.

For the large-scale cultivation of *Aspergillus insuetus* IBT 28485, the 220 plates were extracted with 150 mL EtOAc and 1% formic acid for every 10 plates. The crude extract was fractionated on a diol flash column (Diol, 25 g, 33 mL) using the Isolera One automated flash system. The compounds were eluted with 2 column volumes per fraction: heptane, heptane 1:1 dichloromethane, dichloromethane, dichloromethane 1:1 EtOAc, EtOAc, EtOAc 1:1 MeOH and MeOH. The fractions containing the TMC-120 A and B compounds were fractionated on a reversed-phase C_18_ flash column (15 µm/100 Å, 10 g/15 mL) using the Isolera One flash system. MeOH and Milli-Q water was buffered with 20 mM formic acid and the flow was 15 mL/min. Compounds were eluted with 6 column volumes per fraction: 35% MeOH, 40% MeOH, 42% MeOH, 45% MeOH, 47% MeOH, 50% MeOH, 55% MeOH, 60% MeOH, 70% MeOH, 80% MeOH, and 100% MeOH. TMC-120A and TMC-120B (from fractions 42% MeOH and 45% MeOH) separation was achieved on a Gemini C_6_-Phenyl, 5 μm, 250 × 10 mm column (Phenomenex, Torrance, CA, USA) with a flow of 4 mL/min and using a linear gradient of 40% MeCN in Milli-Q water with 20 mM formic acid going to 70% MeCN in 30 min. TMC- 120C (from fractions 40% MeOH) separation was achieved on a Gemini C_6_-Phenyl, 5 μm, 250 × 10 mm column (Phenomenex, Torrance, CA, USA) with a flow of 4 mL/min and using a linear gradient of 30% MeCN in Milli-Q water going to 60% MeCN in 30 min. Penicisochroman G and ustusorane B (from fractions 70% MeOH and 80% MeOH) separation was achieved on a Gemini C_6_-Phenyl, 5 μm, 250 × 10 mm column (Phenomenex, Torrance, CA, USA) with a flow of 4 mL/min using a linear gradient 65% MeCN in Milli-Q water with 20 mM FA going to 85% MeCN in 30 min. 

TMC-120A: pale yellow solid; ${\left[\alpha \right]}_{D\;}^{20}$-5 (*c* 0.51, MeOH); UV (MeCN) λ_max_: 214 nm, 246 nm, 344 nm, 360 nm; ^13^C-NMR, and ^1^H-NMR in Supplementary Materials. HRESIMS *m/z* 242.1177 [M + H]^+^ (calculated for C_15_H_16_NO_2_, *m/z* 242.1176, ∆ –0.32)

TMC-120B: slightly pale-yellow needles; UV (MeCN) λmax: 215 nm, 239 nm, 262 sh nm, 274 nm, 295 sh nm, 306 nm, 369 nm; ^13^C-NMR, and ^1^H-NMR in Supplementary Materials. HRESIMS *m/z* 240.1019 [M + H]^+^ (calculated for C_15_H_14_NO_2_, *m/z* 240.1019, ∆ 0.14)

### 3.5. Compound and Sample Preparation

For experiments with zebrafish larvae, dry samples and compounds were dissolved in 100% dimethyl sulfoxide (DMSO, spectroscopy grade, Acros Organics (Geel, Belgium)) as 100-fold concentrated stocks and diluted in embryo medium to a final concentration of 1% DMSO content, except for PTZ and valproate which were dissolved in embryo medium (0% DMSO). Of note, for zebrafish exposure to valproate the DMSO content of the embryo medium was adjusted to 1% DMSO in line with other treatments. Control groups were treated with 1% DMSO (VHC) in accordance with the final solvent concentration of tested samples or compounds. For mice experiments, a mixture of polyethylene glycol M.W. 200 (PEG200, >95% purity, Acros Organics (Geel, Belgium)) and 100% DMSO (1:1 PEG200:DMSO) was used as solvent and VHC. Pentylenetetrazole (≥99% purity) and valproate (sodium valproate, ≥98% purity) were purchased from Sigma-Aldrich (Overijse, Belgium).

### 3.6. Experimental Animals

All animal experiments carried out were approved by the Ethics Committee of the University of Leuven (approval numbers 101/2010, 061/2013, 150/2015, 023/2017, and 027/2017) and by the Belgian Federal Department of Public Health, Food Safety & Environment (approval number LA1210199).

#### 3.6.1. Zebrafish

Adult zebrafish (*Danio rerio*) stocks of AB strain (Zebrafish International Resource Center, Oregon, WA, USA) were maintained at 28.0 °C, on a 14/10 h light/dark cycle under standard aquaculture conditions. Fertilized eggs were collected via natural spawning and raised in embryo medium (1.5 mM HEPES, pH 7.2, 17.4 mM NaCl, 0.21 mM KCl, 0.12 mM MgSO_4_, and 0.18 mM Ca(NO_3_)_2_, and 0.6 μM methylene blue) at 28.0 °C, under constant light with regards to the zebrafish PTZ seizure model and under a 14/10-h light/dark cycle with regards to the zebrafish photomotor response assay. 

#### 3.6.2. Mice

Male NMRI mice (weight 18–20 g) were acquired from Charles River Laboratories (France) and housed in polyacrylic cages under a 14/10-h light/dark cycle at 21 °C. The animals were fed a pellet diet and water ad libitum and were allowed to acclimatize for one week before experimental procedures were conducted. Prior to the experiment, mice were isolated in polyacrylic cages with a pellet diet and water ad libitum for habituation overnight in the experimental room, to minimize stress.

### 3.7. Zebrafish Photomotor Response Assay

#### 3.7.1. Behavioral Analysis

The photomotor response of zebrafish embryos was investigated by automated behavioral recording at 30–32 hpf as described before [28]. In the primary screen one replicate well was used per sample tested and each experimental plate contained 6 internal control wells. Each well held 5 embryos that were incubated with VHC (1% DMSO) or sample (1% DMSO, 100 µg/mL) for 2 h prior to behavioral recording. A neuroactive hit was defined as a sample that modified the PMR such that its behavioral fingerprint (16 pseudo Z-scores that together describe the embryonic motion over a 30-s recording period) contained at least one pseudo Z-score with an absolute value equal to or exceeding 5.

#### 3.7.2. Toxicity Evaluation

Each behavioral analysis was followed by visual evaluation of the embryos under a light microscope to assess toxicity of treatment as described before [28]. In brief, overall morphology, heartbeat, and touch response were investigated. MNP samples were scored as being normal or toxic. When embryos showed normal morphology, normal or lowered heartbeat, and normal or lowered touch response the treatment was considered to be normal. In case of an abnormal morphology and/or absence of touch response or heartbeat, a treatment was considered to be toxic.

### 3.8. Zebrafish Pentylenetetrazole Seizure Model

#### 3.8.1. Toxicity Evaluation

The maximum tolerated concentration (MTC) of samples and compounds was determined as described before [7] prior to further experiments and used as the highest test concentration. In case no MTC was reached, the highest soluble concentration or 200 µg/mL was used as the highest test concentration. 

For screening purposes, no MTC was determined, instead behavioral analysis was followed by visual evaluation of the larvae under a light microscope to assess toxicity of treatment. Overall morphology, heartbeat, and touch response were investigated. MNP samples were scored as being normal or toxic. When embryos showed normal morphology, heartbeat, and touch response the treatment was considered to be normal. In case of an abnormal morphology and/or absence of touch response or heartbeat, a treatment was considered to be toxic.

#### 3.8.2. Behavioral Analysis

The locomotion of zebrafish larvae treated with either VHC (1% DMSO) or sample/compound (1% DMSO) for 2 h was investigated by automated behavioral recording at 7 dpf, as described before [7]. In case of the positive control valproate, an 18-h incubation time was used as described in the study of Afrikanova and colleagues [32]. Larval behavior was depicted as mean actinteg units per 5 min during the 30-min recording period and over consecutive time intervals. Data are expressed as mean ±SEM for single experiments and for independent experiments of which the means or raw data are pooled. 

In the first secondary screen three replicate wells were used per sample (100 µg/mL) tested and each experimental plate contained 12 internal control wells. In the second secondary screen 6 replicate wells were used per sample and concentration tested (100, 33, and 11 µg/mL), again 12 internal control wells were used per experimental plate.

#### 3.8.3. Electrophysiology

Non-invasive LFP recordings were measured from the midbrain (optic tectum) of 7-dpf zebrafish larvae pre-incubated with VHC only, PTZ only, compound and VHC, or compound and PTZ. Larvae were incubated for approximately 2 h with VHC (1% DMSO) or test compound (1% DMSO) in a 100 µL volume (in case of the positive control valproate, an 18-h incubation time was used as described in the study of Afrikanova and colleagues [32]), whereafter, an equal volume of VHC (embryo medium) or 40 mM PTZ (20 mM working concentration) was added for 15 min prior to recording. These steps occurred at 28 °C, while further manipulation and electrophysiological recordings occurred at room temperature (21 °C) and were performed as described before [7]. Each recording lasted 600 s. Manual analysis was completed by quantification of the number and duration of epileptiform-like events with Clampfit 10.2 software (Molecular Devices Corporation, San Jose, CA, USA). An electrical discharge was considered epileptiform if it was a polyspiking event comprising at least 3 spikes with a minimum amplitude of three times the baseline amplitude and a duration of at least 100 ms. Data are expressed as mean ±SD.

### 3.9. Mouse 6-Hz (44 mA) Psychomotor Seizure Model

The antiseizure activity of compounds was investigated in the mouse 6-Hz (44 mA) psychomotor seizure model as described before [7]. In brief, 50 µL (injection volume was adjusted to the individual weight) of VHC (PEG200:DMSO 1:1) or treatment (an ASD or test compound dissolved in VHC) was i.p. injected in NMRI mice (average weight 30 g, range 26–36 g) and after 30 min psychomotor seizures were induced by corneal electrical stimulation (6 Hz, 0.2 ms rectangular pulse width, 3 s duration, 44 mA) using an ECT Unit 5780 (Ugo Basile, Comerio, Italy). Seizure durations were measured during the experiment by experienced researchers, familiar with the different seizure behaviors. In addition, seizure durations were determined by blinded video analysis to confirm or correct the initial observations. Data are expressed as mean ±SD.

## 4. Conclusions

Despite the well-appreciated potential of marine natural products for drug discovery, and the high need for new ASDs, to the best of our knowledge no large-scale ASD discovery has been done so far with MNPs. The PharmaSea project provided the consortium with the framework to perform marine ASD discovery from more than 2000 crude extracts and fractions from microbial species harvested across oceans worldwide. The use of zebrafish embryos and larvae enabled in vivo antiseizure analysis using only 10 µg per well per screening round. Thus, in our zebrafish-based screening approach the issue of low quantities typically available for initial bioactivity analysis was addressed without compromising on the value of an in vivo read-out. 

This study describes the zebrafish-based discovery of antiseizure activity of a fraction (hit SK0107) of the crude extract of the marine-derived fungus *Aspergillus insuetus* and the bioactivity-guided isolation of its active constituents TMC-120A and TMC-120B, which we propose as lead compounds worth further investigation for the treatment of epileptic seizures. Not only did both compounds show antiseizure activity in the zebrafish PTZ seizure model, they also ameliorated the hyperexcitable state of the zebrafish brain due to pronounced anti-epileptiform activity. These results also translated to a mouse model of drug-resistant focal seizures. The structural analogues TMC-120C, penicisochroman G, and ustusorane B were isolated based on their structural similarity to TMC-120A and TMC-120B and showed antiseizure activity in the zebrafish PTZ seizure model. Continued antiseizure characterization will demonstrate the potential of these novel antiseizure compounds in the treatment of epilepsy. Importantly, with this study PharmaSea shows again that the marine environment is a valuable resource in the search for novel ASD candidates and that the zebrafish model is a helpful tool in this ongoing search.

## 5. Patents

A patent application (PCT/EP2018/073149) was filed on 28th of August 2018 with regards to the research findings described in this study.

## Figures and Tables

**Table 1:** Potential TMC-120A and TMC-120B producing strains from *Aspergillus* section *Usti*.

IBT Number	Species
4133	*Aspergillus ustus*
10619	*Aspergillus ustus*
28485	*Aspergillus insuetus*
914826	*Aspergillus calidoustus*

Closely related species belonging to *Aspergillus* section *Usti* from the IBT culture collection at the Department of Biotechnology and Biomedicine (Technical University of Denmark, Kgs. Lyngby, Denmark) that are potential TMC-120A and TMC-120B producing strains.

## Supplementary Materials

The following are available online at https://www.mdpi.com/1660-3397/17/11/607/s1, Figure S1: ^1^H-NMR (800 MHz CDCl_3_) of TMC-120A, Figure S2: ^13^C-NMR (200 MHz CDCl_3_) of TMC-120A, Figure S3: ^1^H-NMR (800 MHz CDCl_3_) of TMC-120B, Figure S4: ^13^C-NMR (200 MHz CDCl_3_) of TMC-120B, Table S1: NMR spectroscopic data for TMC-120B, Table S2: NMR spectroscopic data for TMC-120A and TMC-120B, Table S3: HRMS, UV/Vis, optical rotations, and NMR spectroscopic data for penicisochroman G, ustusorane B, and TMC-120C, Figures S5–S7: ^1^H-NMR spectra of TMC-120C, ustusorane B, and penicisochroman G, Figures S8 and S9: Behavioral and electrophysiological antiseizure analysis of positive control valproate in the zebrafish PTZ seizure model.
