# Supplementary material for: Zebrafish-Based Discovery of Antiseizure Compounds from the North Sea: Isoquinoline Alkaloids TMC-120A and TMC-120B
Source: Mar Drugs. 2019 Oct 25;17(11):607. doi: 10.3390/md17110607 (PMC6891649; doi:10.3390/md17110607)
Supplement: Supplementary file 1 [file marinedrugs-17-00607-s001.pdf]

marine drugs

*Article*

# Supporting Information

# Zebrafish-Based Discovery of Antiseizure Compounds from the North Sea: Isoquinoline Alkaloids TMC-120A and TMC-120B

**Daniëlle Copmans [1,†], Sara Kildgaard [2,†], Silas A. Rasmussen [2], Monika Ślęzak [1], Nina Dirkx [1], Michèle Partoens [1], Camila V. Esguerra [1,3], Alexander D. Crawford [1,4], Thomas O. Larsen [2,*] and Peter A. M. de Witte [1,*]**

Page 2. Table S1. NMR spectroscopic data for TMC-120B

Page 2. Table S2. NMR spectroscopic data for TMC-120A and TMC-120B

Page 3. Figure S1. $^{1}$H-NMR (800 MHz $CDCl_3$) of TMC-120A

Page 3. Figure S2. $^{13}$C-NMR (200 MHz $CDCl_3$) of TMC-120A

Page 4. Figure S3. $^{1}$H-NMR (800 MHz $CDCl_3$) of TMC-120B

Page 4. Figure S4. $^{13}$C-NMR (200 MHz $CDCl_3$) of TMC-120B

Page 5. Table S3. HRMS, UV/Vis, optical rotations, and NMR spectroscopic data for penicisochroman G, ustusorane B, and TMC-120C

Page 6. Figure S5. $^{1}$H-NMR (800 MHz $CDCl_3$) of TMC-120C

Page 6. Figure S6. $^{1}$H-NMR (600 MHz $CDCl_3$) of ustusorane B

Page 7. Figure S7. $^{1}$H-NMR (600 MHz $CDCl_3$) of penicisochroman G

Page 7. Figure S8. Behavioral antiseizure analysis of positive control valproate in the zebrafish PTZ seizure model

Page 8. Figure S9. Electrophysiological antiseizure analysis of positive control valproate in the zebrafish PTZ seizure model

**Table S1.** NMR spectroscopic data for TMC-120B.

| TMC-120B | | |
|---|---|---|
| **Position** | **$\delta_H$ (mult, *J*)** | **$\delta_C$** |
| 1 | - | - |
| 2 | - | 145.6 |
| 3 | - | 182.1 |
| 3a | - | 119.3 |
| 4 | 7.81 d(8.6) | 124.4 |
| 5 | 7.36 d(8.6) | 120.8 |
| 5a | - | 141.3 |
| 6 | 7.53 s | 119.8 |
| 7 | - | 156.7 |
| 8 | - | - |
| 9 | 9.55 s | 146.3 |
| 9a | - | 114.6 |
| 9b | - | 164.0 |
| 10 | 2.74 s | 24.9 |
| 11 | - | 133.7 |
| 12 | 2.42 s | 17.6 |
| 13 | 2.24 s | 20.5 |

NMR spectroscopic data ($^1$H NMR data were obtained at 400 MHz in $CDCl_3$ and $^{13}$C data were obtained at 100 MHz in $CDCl_3$, δ in ppm, *J* in Hz) for TMC-120B isolated from the crude extract of *Aspergillus insuetus* IBT 28443.

**Table S2.** NMR spectroscopic data for TMC-120A and TMC-120B.

| . | TMC-120A | | TMC-120B | |
|---|---|---|---|---|
| **Position** | **$\delta_H$ (mult, *J*)** | **$\delta_C$** | **$\delta_H$ (mult, *J*)** | **$\delta_C$** |
| 1 | - | - | - | - |
| 2 | 4.85 d(4.0) | 91.8 | - | 145.8 |
| 3 | - | 199.8 | - | 182.4 |
| 3a | - | 117.8 | - | 119.6 |
| 4 | 7.71 d(8.6) | 124.3 | 7.80 d(8.6) | 124.4 |
| 5 | 7.30 d(8.6) | 120.3 | 7.36 d(8.6) | 120.8 |
| 5a | - | 142.5 | - | 141.5 |
| 6 | 7.53 s | 119.9 | 7.53 s | 119.8 |
| 7 | - | 157.4 | - | 156.8 |
| 8 | - | - | - | - |
| 9 | 9.55 s | 146.6 | 9.54 s | 146.4 |
| 9a | - | 115.2 | - | 114.8 |
| 9b | - | 174.1 | - | 164.2 |
| 10 | 2.75 s | 24.7 | 2.74 s | 24.9 |
| 11 | 2.46 m | 31.3 | - | 134.0 |
| 12 | 0.92 d(6.9) | 15.9 | 2.42 s | 17.8 |
| 13 | 1.23 d(6.9) | 19.0 | 2.24 s | 20.6 |

NMR spectroscopic data ($^1$H NMR data were obtained at 800 MHz in $CDCl_3$ and $^{13}$C data were obtained at 200 MHz in $CDCl_3$, δ in ppm, *J* in Hz) for TMC-120A and TMC-120B isolated from the crude extract of *Aspergillus insuetus* IBT 28485.

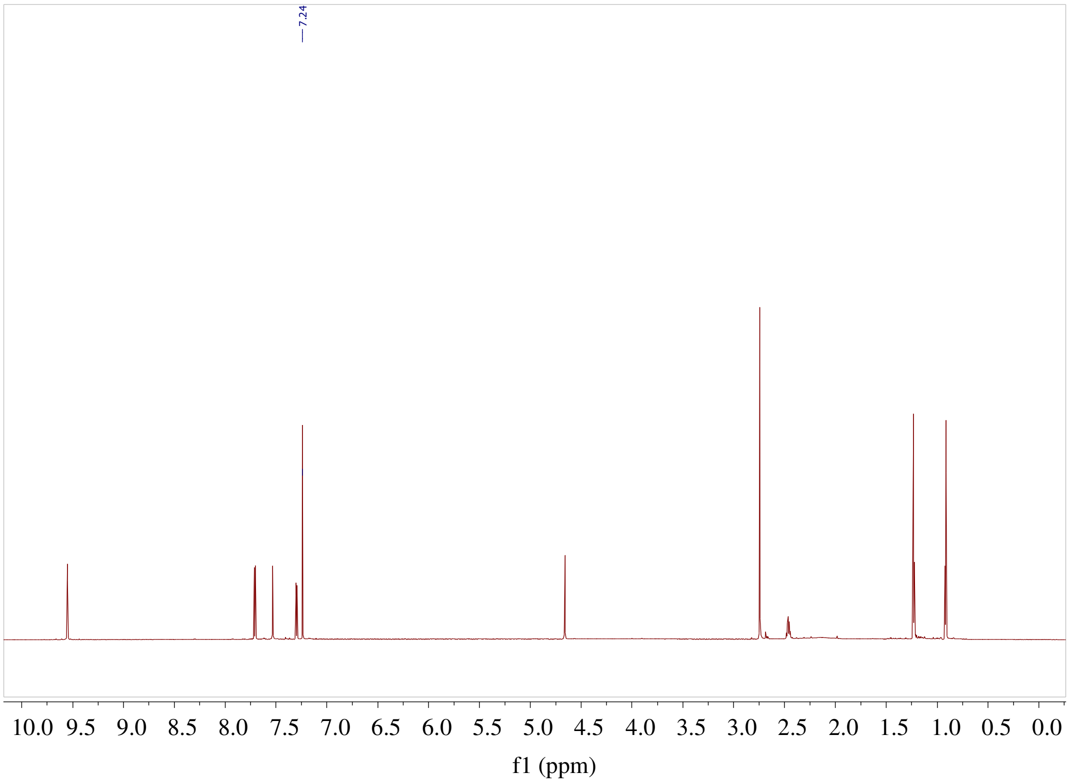


**Figure S1.** $^{1}$H-NMR (800 MHz $CDCl_3$) of TMC-120A.

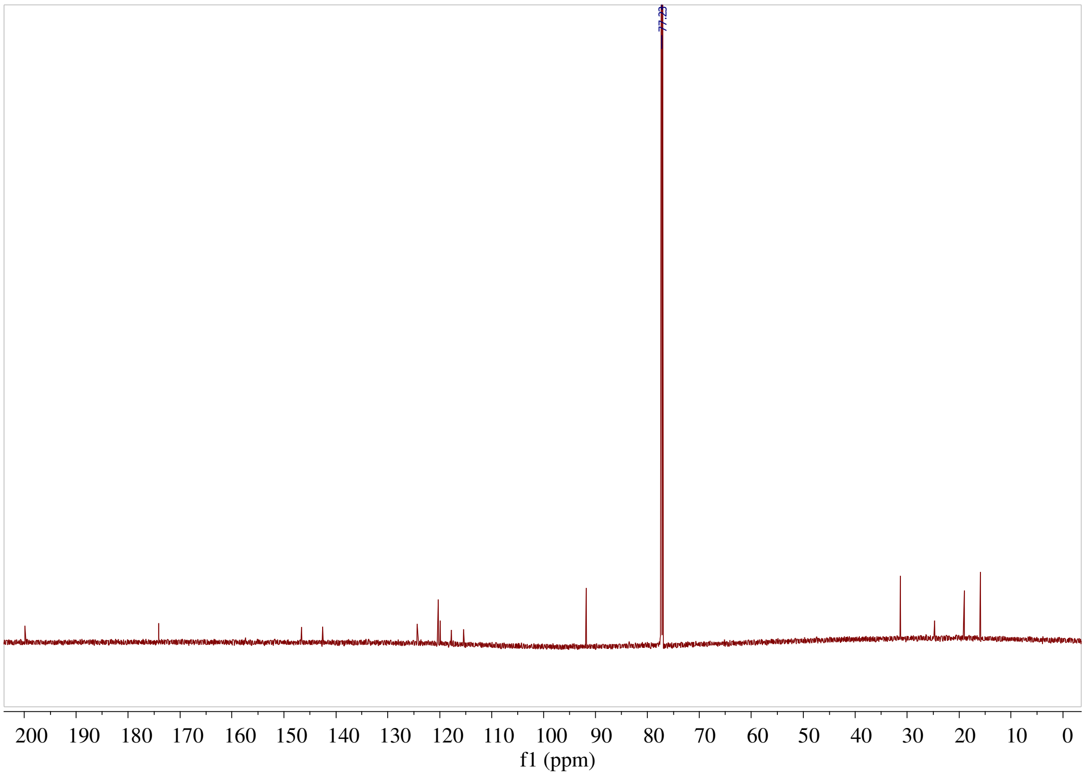


**Figure S2.** $^{13}$C-NMR (200 MHz $CDCl_3$) of TMC-120A.

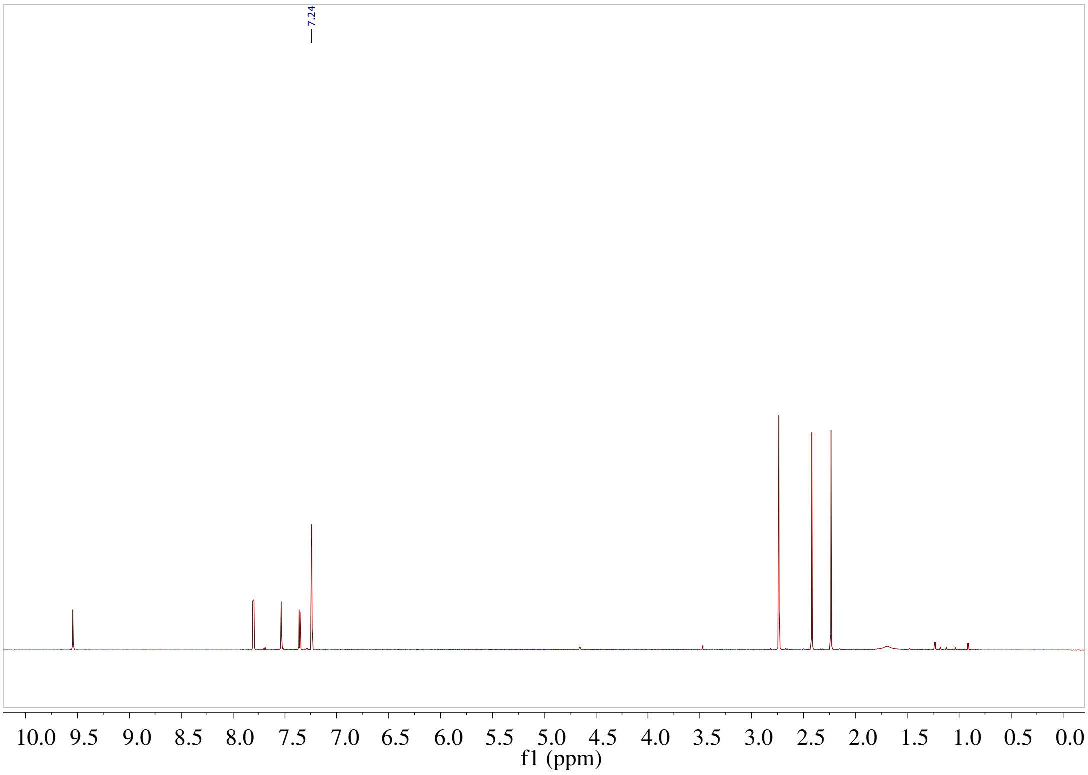


**Figure S3.** $^{1}$H-NMR (800 MHz $CDCl_3$) of TMC-120B.

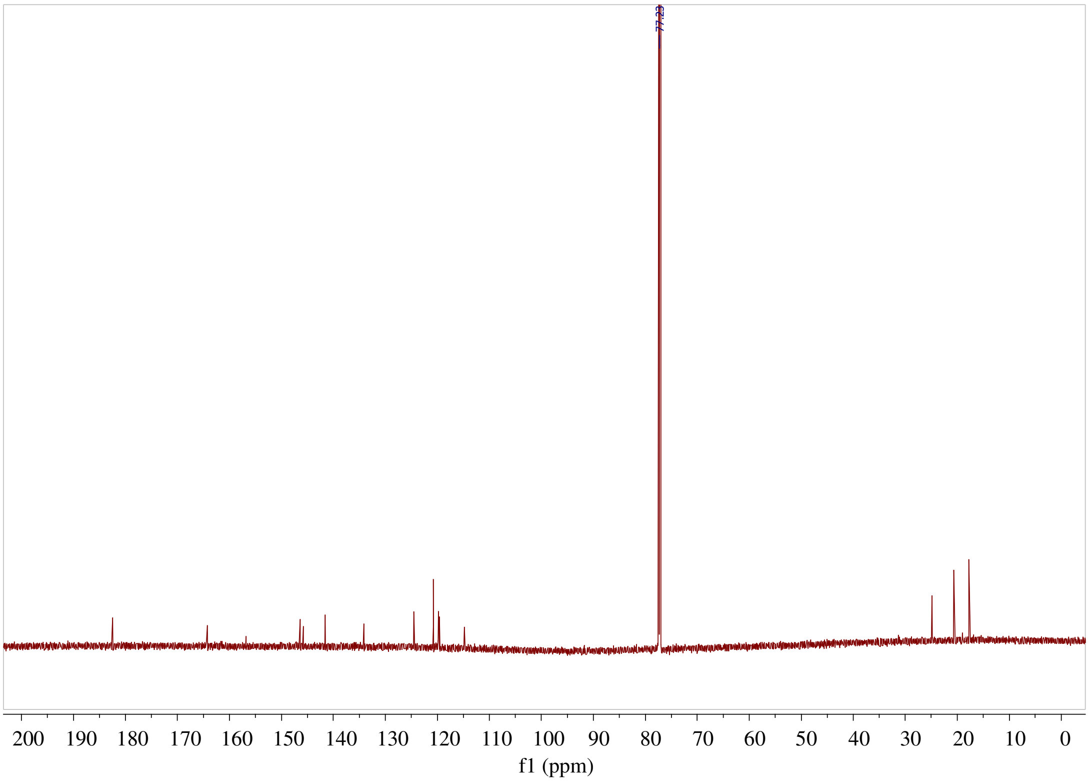


**Figure S4.** $^{13}$C-NMR (200 MHz $CDCl_3$) of TMC-120B.

**Table S3.** HRMS, UV/Vis, optical rotations, and NMR spectroscopic data for penicisochroman G, ustusorane B, and TMC-120C.

| | **Penicisochroman G** | | **Ustusorane B** | | **TMC-120C** | |
|---|---|---|---|---|---|---|
| **Position** | **$\delta_H$ (mult, *J*)** | **$\delta_C$** | **$\delta_H$ (mult, *J*)** | **$\delta_C$** | **$\delta_H$ (mult, *J*)** | **$\delta_C$** |
| 1 | - | - | - | - | - | - |
| 2 | 4.39 d(3.9) | 90.4 | - | 144.6 | - | 110.3 |
| 3 | - | 200.6 | - | 182.2 | - | 198.7 |
| 3a | - | 120.0 | - | 120.7 | - | 115.8 |
| 4 | 7.43 d(8.0) | 123.9 | 7.52 d(8.0) | 122.9 | 7.52 d(8.2) | 124.5 |
| 5 | 6.57 d(8.0) | 117.0 | 6.63 d(8.0) | 116.2 | 6.99 d(8.2) | 119.6 |
| 5a | - | 142.0 | - | 139.6 | - | 142.1 |
| 6 | 5.65 s | 101.7 | 5.67 s | 100.7 | 7.14 s | 119.6 |
| 7 | - | 160.0 | - | 158.5 | - | 156.7 |
| 8 | - | - | - | - | - | - |
| 9 | 5.20 d(13.2)<br>5.25 d(13.2) | 62.8 | 5.26 s | 61.5 | 9.46 s | 146.8 |
| 9a | - | 108.9 | - | 107.7 | - | 114.7 |
| 9b | - | 168.3 | - | 158.9 | - | 171.6 |
| 10 | 1.95 s | 20.1 | 1.95 s | 18.7 | 2.50 s | 23.9 |
| 11 | 2.31 m | 31.2 | - | 129.9 | 2.40 m | 34.1 |
| 12 | 0.84 d(6.9) | 15.8 | 2.33 s | 16.2 | 0.96 | 16.1 |
| 13 | 1.13 d(6.9) | 19.0 | 2.06 s | 19.0 | 1.21 d(6.6) | 15.7 |

**TMC-120C**: slightly pale yellow needles; $[\alpha]_D^{20}$-11 (*c* 0.14, MeOH); UV (MeCN) $\lambda_{max}$: 215 nm, 250 nm, 359 nm; HRESIMS *m*/*z* 258.1123 [M+H]$^+$ (calculated for $C_{15}H_{16}NO_3$, *m*/*z* 258.1125, Δ 0.57).

**penicisochroman G**: yellow solid; $[\alpha]_D^{20}$+13 (*c* 0.20, MeOH); UV (MeCN) $\lambda_{max}$: 213 nm, 255 nm, 355 nm; HRESIMS *m*/*z* 245.1171 [M+H]$^+$ (calculated for $C_{15}H_{17}O_3$, *m*/*z* 240.1172, Δ 0.5).

**ustusorane B**: yellow solid; UV (MeCN) $\lambda_{max}$: 225 nm, 278 nm, 287 nm, 368 nm; HRESIMS *m*/*z* 243.1014 [M+H]$^+$ (calculated for $C_{15}H_{15}O_3$, *m*/*z* 243.1016, Δ 0.7).

NMR spectroscopic data for penicisochroman G and ustusorane B ($^1$H NMR data were obtained at 600 MHz in $CDCl_3$ and $^{13}$C data were obtained at 150 MHz in $CDCl_3$, δ in ppm, *J* in Hz) and TMC-120C ($^1$H NMR data were obtained at 800 MHz in $CDCl_3$ and $^{13}$C data were obtained at 200 MHz in $CDCl_3$, δ in ppm, *J* in Hz) isolated from the crude extract of *Aspergillus insuetus* IBT 28485. The $^{13}$C-chemical shifts have been determined using the HSQC and HMBC spectra.

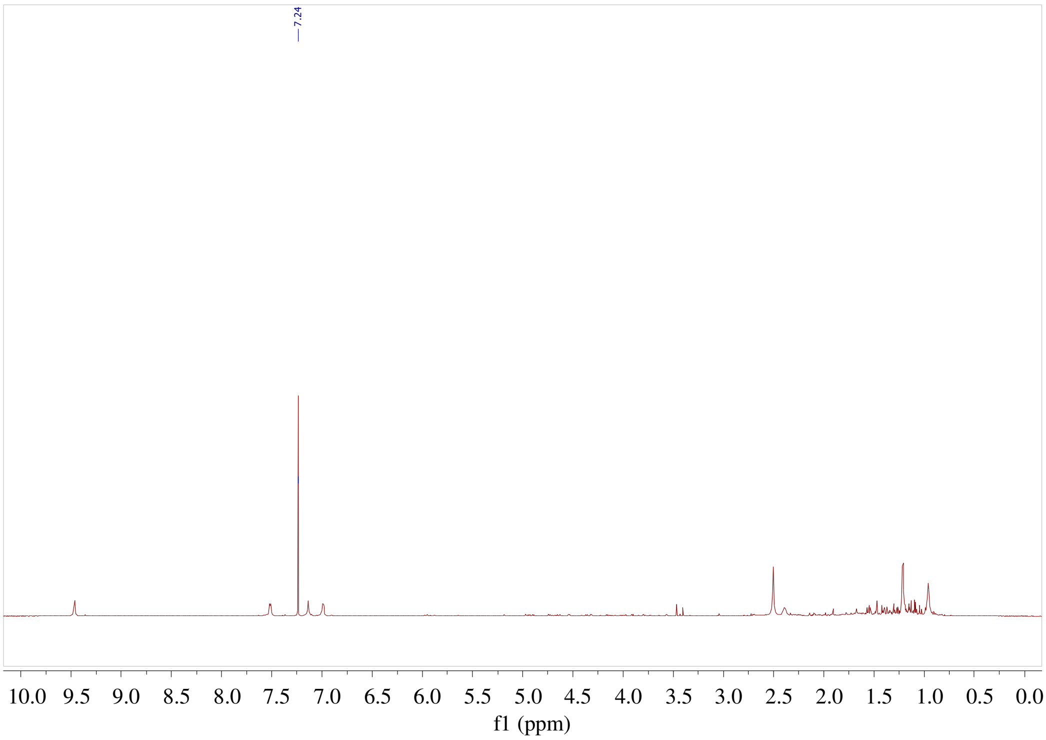


**Figure S5.** $^{1}$H-NMR (800 MHz $CDCl_3$) of TMC-120C.

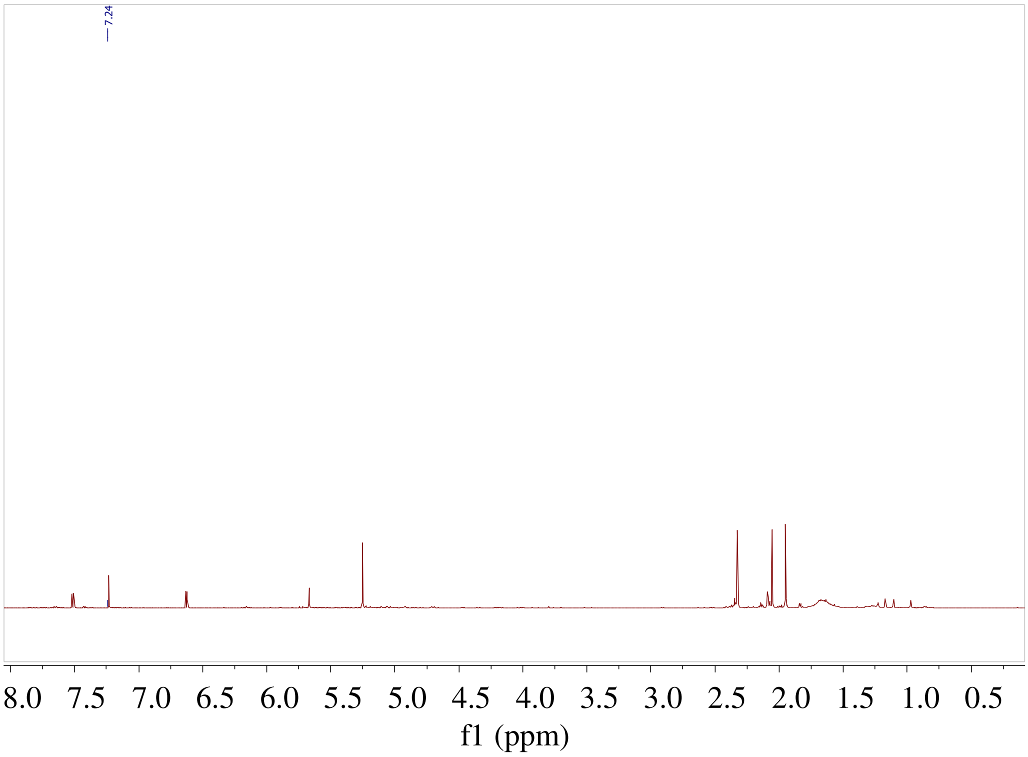


**Figure S6.** $^{1}$H-NMR (600 MHz $CDCl_3$) of ustusorane B.

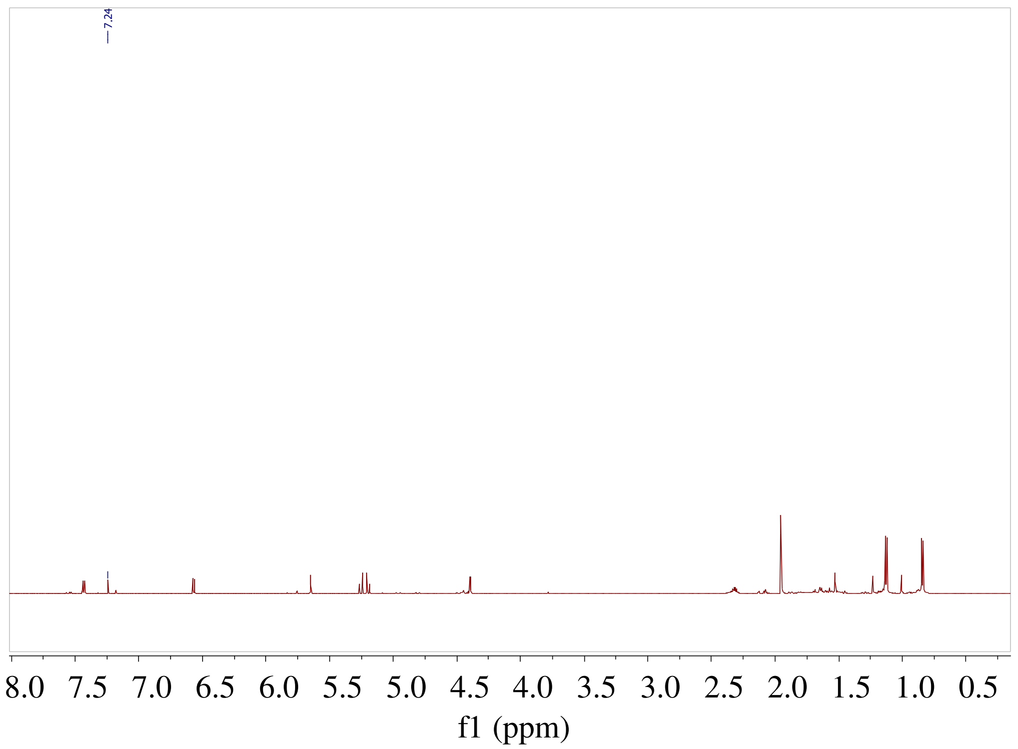


**Figure S7.** $^{1}$H-NMR (600 MHz $CDCl_3$) of penicisochroman G.

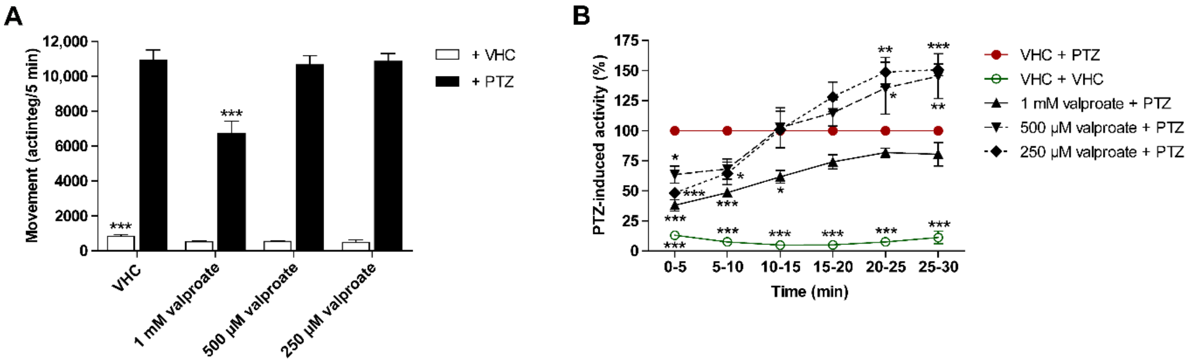


**Figure S8.** Behavioral antiseizure analysis of positive control valproate in the zebrafish PTZ seizure model. Antiseizure activity of valproate in the zebrafish pentylenetetrazole (PTZ) seizure model after 18 h of incubation at its maximum tolerated concentration (MTC), MTC/2, and MTC/4. PTZ-induced seizure-like behavior is expressed as mean actinteg units per 5 min (±SEM) during the 30-min recording period (**A**) and over consecutive time intervals (**B**). Means are pooled from three independent experiments with each 9-10 replicate wells per condition. Statistical analysis: (**A**) one-way ANOVA with Dunnett's multiple comparison test, (**B**) two-way ANOVA with Bonferroni posttests (GraphPad Prism 5, San Diego, CA, USA). Significance levels: * $p \leq 0.05$; ** $p \leq 0.01$; *** $p \leq 0.001$. Abbreviation: vehicle, VHC.

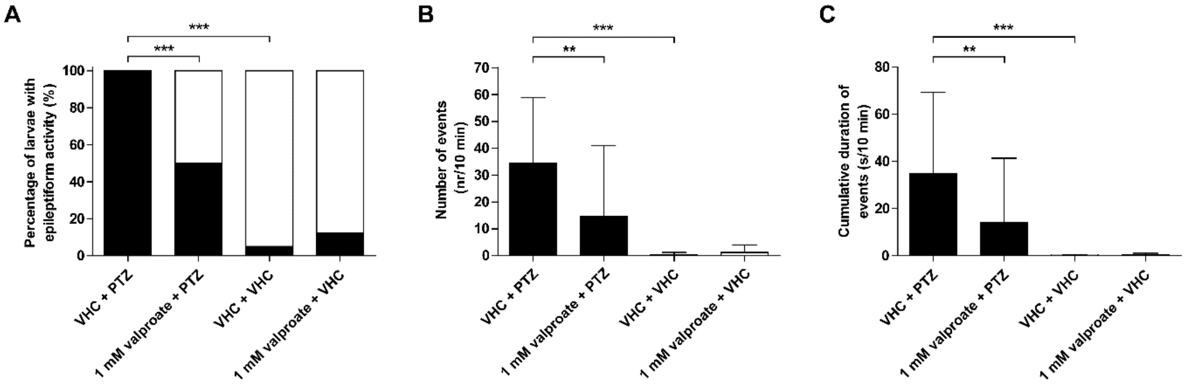


**Figure S9.** Electrophysiological antiseizure analysis of positive control valproate in the zebrafish PTZ seizure model. Noninvasive local field potential recordings from the optic tectum of larvae pre-exposed to vehicle (VHC) and pentylenetetrazole (PTZ), VHC only, valproate and PTZ, or valproate and VHC. Larvae were incubated with either 1 mM valproate or VHC for 18 h, conform with the maximum tolerated concentration and incubation time of valproate used in the behavioral assay. Larvae are considered to possess epileptiform brain activity when three or more epileptiform events occurred during a 10-min recording (**A**). Epileptiform discharges are quantified by the number (mean ±SD) (**B**) and cumulative duration (mean ±SD) (**C**) of events per 10-min recording. Number of replicate wells per condition: 19 larvae were used for control conditions (VHC + PTZ and VHC + VHC groups), 18 larvae were used for the condition valproate + PTZ, and 16 larvae were used for the condition valproate + VHC. Statistical analysis: (**A**) Fisher's exact test with Bonferroni posttest, (**B**, **C**) Kruskal-Wallis test with Dunn's multiple comparison test (data did not pass the Shapiro-Wilk normality test) (GraphPad Prism 5, San Diego, CA, USA). Significance levels: * $p \leq 0.05$; ** $p \leq 0.01$; *** $p \leq 0.001$.

© 2019 by the authors. Licensee MDPI, Basel, Switzerland. This article is an open access article distributed under the terms and conditions of the Creative Commons Attribution (CC BY) license (http://creativecommons.org/licenses/by/4.0/).
